# Biological Calibration for Web-Based Hearing Tests: Evaluation of the Methods

**DOI:** 10.2196/jmir.2798

**Published:** 2014-01-15

**Authors:** Marcin Masalski, Tomasz Grysiński, Tomasz Kręcicki

**Affiliations:** ^1^Department and Clinic of Otolaryngology, Head and Neck SurgeryWroclaw Medical UniversityWrocławPoland; ^2^Institute of Biomedical Engineering and InstrumentationWroclaw University of TechnologyWrocławPoland

**Keywords:** pure-tone audiometry, computer-assisted instruction, self-examination

## Abstract

**Background:**

Online hearing tests conducted in home settings on a personal computer (PC) require prior calibration. Biological calibration consists of approximating the reference sound level via the hearing threshold of a person with normal hearing.

**Objective:**

The objective of this study was to identify the error of the proposed methods of biological calibration, their duration, and the subjective difficulty in conducting these tests via PC.

**Methods:**

Seven methods have been proposed for measuring the calibration coefficients. All measurements were performed in reference to the hearing threshold of a normal-hearing person. Three methods were proposed for determining the reference sound level on the basis of these calibration coefficients. Methods were compared for the estimated error, duration, and difficulty of the calibration. Web-based self-assessed measurements of the calibration coefficients were carried out in 3 series: (1) at a otolaryngology clinic, (2) at the participant’s home, and (3) again at the clinic. Additionally, in series 1 and 3, pure-tone audiometry was conducted and series 3 was followed by an offline questionnaire concerning the difficulty of the calibration. Participants were recruited offline from coworkers of the Department and Clinic of Otolaryngology, Wroclaw Medical University, Poland.

**Results:**

All 25 participants, aged 22-35 years (median 27) completed all tests and filled in the questionnaire. The smallest standard deviation of the calibration coefficient in the test-retest measurement was obtained at the level of 3.87 dB (95% CI 3.52-4.29) for the modulated signal presented in accordance with the rules of Bekesy’s audiometry. The method is characterized by moderate duration time and a relatively simple procedure. The simplest and shortest method was the method of self-adjustment of the sound volume to the barely audible level. In the test-retest measurement, the deviation of this method equaled 4.97 dB (95% CI 4.53-5.51). Among methods determining the reference sound level, the levels determined independently for each frequency revealed the smallest error. The estimated standard deviations of the difference in the hearing threshold between the examination conducted on a biologically calibrated PC and pure-tone audiometry varied from 7.27 dB (95% CI 6.71-7.93) to 10.38 dB (95% CI 9.11-12.03), depending on the calibration method.

**Conclusions:**

In this study, an analysis of biological calibration was performed and the presented results included calibration error, calibration time, and calibration difficulty. These values determine potential applications of Web-based hearing tests conducted in home settings and are decisive factors when selecting the calibration method. If there are no substantial time limitations, it is advisable to use Bekesy method and determine the reference sound level independently at each frequency because this approach is characterized by the lowest error.

## Introduction

Sound systems of modern home electronic equipment, such as a personal computer (PC), tablet, or smartphone, offer opportunities to conduct hearing examinations at low cost and on a large scale [1-4]. The population of people who are computer literate is aging and their hearing sensitivity is declining. Therefore, the number of individuals potentially interested in this type of testing is increasing. Additionally, research shows that the use of the Internet is higher in the hearing-impaired population in comparison to similar age groups in the general population [5,6].

Hearing tests conducted remotely in home settings on PCs can be divided into 2 groups depending on the necessity of conducting prior calibration. The examinations which do not require prior calibration are usually screening tests represented by speech-in-noise tests [1,7-9]. The speech-in-noise test involves the evaluation of speech intelligibility in relation to signal-to-noise ratio; therefore, the knowledge of the absolute sound level is not required. The speech-in-noise test contributes to increased identification of hearing loss [1] and is more useful in screening tests than a short questionnaire [7]. Additionally, sensitivity of the test can be improved after applying low-band noise [9].

However, most hearing tests, including the basic examination in the form of pure-tone audiometry, require prior calibration of the system, and its omission leads to significant measurement errors [10]. Calibration consists of determining the reference sound level. For the purposes of the hearing test conducted in in-home conditions, it can be performed in a number of ways. The calibration of a PC system can be carried out in a laboratory setting beforehand and then later used for home-based examinations [11]. Another solution is to prepare software that will cooperate with an audio set whose parameters are known, consisting of a sound card and headphones [12]. In this case, to conduct a home-based examination requires purchasing a particular set. Both the previously mentioned solutions limit accessibility of the hearing test because they require efforts that are unjustified in the case of a single hearing test. In light of this, biological calibration seems a sensible solution, consisting of approximation of the reference sound level by the hearing threshold of a person with normal hearing. Usually the reference sound level is assumed at 0 decibel hearing level (dB HL).

Honeth et al [3] used biological calibration based on evaluation of the hearing threshold of a person with normal hearing at the following frequencies: 500 Hz, 1 kHz, 2 kHz, 6 kHz, and 8 kHz. The task of the reference person was to set the volume marker at the level at which the sound was barely audible. In this way, the reference sound level was determined individually for each frequency. The test results were compared with pure-tone audiometry and exhibited the greatest error at 2 and 4 kHz, corresponding to 5.6 dB (SD 8.29) and 5.1 dB (SD 6.9), respectively. In all, 89% of the tests were conducted on the same computer and with the same reference person. Masalski and Kręcicki [4] also used biological calibration based on evaluation of the hearing threshold of a reference person by using a volume marker. Calibration was conducted for 1 kHz only, and the values of 0 dB HL at other frequencies were calculated on the basis of the A-weight filter. Self-examinations conducted by the participants on their home computers calibrated by their normal-hearing family members showed a mean error of the hearing threshold compared to pure-tone audiometry at the level of -1.35 dB (SD 10.66).

The error analysis of the pure-tone audiometry conducted on a PC calibrated by the biological method showed significant influence of the calibration error [4]. The standard deviation of the calibration error at 1 kHz was 6.19 dB, whereas the measurement was additionally burdened with an estimation error of 0 dB HL conducted on the basis of the A-weight filter at other frequencies. The highest estimation error was at 250 Hz at the level of 7.28 dB. Nevertheless, sensitivity and specificity values calculated for the detection of noise-induced hearing loss, compared with pure-tone audiometry, were found to be reasonable (ie, at the level of sensitivity 0.89, 95% CI 0.74-1.0 and specificity 0.89, 95% CI 0.76-1.0). Similar sensitivity and specificity were obtained by Honeth et al [3] (sensitivity 0.75, 95% CI 0.51-0.90 and specificity 0.96, 95% CI 0.96-0.99).

The application of pure-tone audiometry based on biological calibration depends significantly on the measurement error. Because of the much larger error of biological calibration than tolerance required by the standards (ie, ±3 dB in the frequency range 125 Hz to 5 kHz [13]), the home test cannot be an alternative to classical pure-tone audiometry. However, it may be applied as a screening test as well as in other situations suggested in other studies [3,4], such as self-monitoring of hearing for some disorders (eg, fluctuating hearing loss, tinnitus, sudden deafness, otosclerosis, Ménière’s disease), during treatment with ototoxic drugs, in large-scale epidemiological studies, in cases of limited access to specialist equipment (eg, at the general practitioner’s office or in countries with low economic status), and also as a telemedical examination combined with a questionnaire to determine the direction of further treatment. However, before verification of these applications it is advisable to optimize biological calibration [4].

This paper presents 7 methods of measuring the calibration coefficients. All measurements were performed in reference to the hearing threshold of a normal-hearing person. For each method, the measurement error was determined, as well as the timeframe for its calibration and the difficulty level. Next, 3 methods were proposed for determining the reference sound level on the basis of these calibration coefficients and an error analysis was conducted for each.

## Methods

### Overview

The proposed methods of biological calibration consist in measuring the calibration coefficient that describes the threshold sound level of the reference person. Seven calibration methods were proposed: (1) calibration using an amplitude-modulated signal, (2) calibration using 2 sounds differing by 5 dB, (3) calibration using 2 sounds differing by 2 dB, (4) the ascending method with a 5-dB step, (5) the ascending method with a 2-dB step, (6) calibration based on Bekesy audiometry using the continuous signal, and (7) calibration based on Bekesy audiometry using an amplitude-modulated signal. In methods 1-5, the assessment was conducted for the following frequencies: 125 Hz, 500 Hz, 1 kHz, 2 kHz, 4 kHz, 6 kHz, and 8 kHz. In methods 6 and 7, the frequency was changed in a continuous way from 62.5 Hz to 16 kHz. The sound signal was presented bilaterally.

### Amplitude-Modulated Signal Method

In the calibration with amplitude-modulated signal (method 1), the presented signal was amplitude-modulated using rectangular envelope with frequency of 1 Hz and modulation depth of 100%. The task of the reference person was to set the volume marker in such a way that the generated sound be barely audible. The step of the volume marker was 1 dB.

### Dual Tone Methods

During calibration with 2 sounds differing in intensity (methods 2 and 3), 2 tone signals with a given frequency and a duration of 1 s were presented in turns. The task of the reference person was to set the volume marker in such a way that the louder of the 2 sounds was still audible, and the quieter inaudible. In method 2, the signals differed by 5 dB, whereas in method 3, the difference was 2 dB. The step of the volume marker was 1 dB.

### Ascending Methods

The ascending method (methods 4 and 5) was based on the ascending algorithm used for the assessment of the hearing threshold in pure-tone audiometry [14]. A signal with a given frequency was presented for a random duration from 2 s to 7 s. The task of the reference person was to press a button on hearing a sound and release it when it was no longer audible. The button should be pressed up to 2 s from the start of playing the sound and released up to 2 s after it stopped. The level of the tone was reduced in 10-dB steps until no further response occurred, and then it was increased in 5-dB steps until the participant responded. The calibration coefficient was defined as the lowest level at which responses occurred in at least half of the series of ascending trials with a minimum of 2 responses required at that level. No more than 5 previously conducted ascending trials were taken into account. Method 4 used 5-dB and 10-dB steps. Method 5 used a 4-dB step down and a 2-dB step up.

### Bekesy Methods

During calibrations based on Bekesy audiometry, the frequency of a presented signal was increased at the speed of 1 octave/60 s, simultaneously with the change of its intensity. The task of the reference person was to press a button on hearing the signal and keep it pressed for as long as the sound was audible. The intensity of the sound was reduced at a speed of 2 dB/s when the sound was audible, and increased at the same speed when the sound was inaudible. The value of the calibration coefficient was determined as the mean of the values of the sound intensity at which a change in the status of the button occurred, after rejecting the outliers on the basis of the Grubbs’ test [15]. Coefficients were determined for the frequencies of 125 Hz, 500 Hz, 1 kHz, 2 kHz, 4 kHz, 6 kHz, and 8 kHz by calculating the mean of the range ±0.5 octave. Method 6 used a continuous signal, whereas method 7 used a signal modulated in amplitude by a sinusoidal envelope with frequency of 2 Hz and modulation depth of 100%.

All 7 methods were implemented in Java technology in the form of applets embedded in a Web browser. The calibration coefficients expressing the sound intensity in decibels, together with the duration of examinations, were recorded in the database. On completion of all the tests, the participant filled in an offline questionnaire (Multimedia Appendix 1) on the difficulty of the tests by assigning each method a value from 0 (the easiest method) to 10 (the hardest method).

### Reference Sound Level Methods

In addition to the 7 methods of measuring the calibration coefficients, 3 methods were proposed for determining the reference sound level: (1) the reference sound level determined independently for each frequency depending on the value of calibration coefficient measured at this frequency, (2) the reference sound level estimated by a model fitted to calibration coefficients determined at all frequencies, and (3) as (2) except the model was fitted to a single coefficient determined at the frequency characterized by the smallest measurement error.

Participants were recruited offline from coworkers of Department and Clinic of Otolaryngology, Wroclaw Medical University, Poland, using face-to-face prompting from September 2012 to March 2013. The eligibility criteria were age younger than 35 years, lack of previous hearing problems, owning headphones and a PC at home and basic skills to operate it, and the willingness to participate in the research. Each participant performed calibration using all 7 methods 3 times. In series 1, the study was carried out in a sound booth with the use of notebook Dell Vostro 1310 with Microsoft Windows 7 operational system and Technics RP-F290 headphones; in series 2, each person was asked to perform calibration on their own home computer using their own headphones in the quietest conditions possible, preferably late in the evening or at night to minimize background noise level and to create conditions close to those in the sound booth; and series 3 was the repetition of examinations from series 1. Because of the relatively long duration of the series, the participants were informed about the option of taking a break when they felt tired, and most of the participants took advantage of this. In series 1 and 3, pure-tone audiometry was performed with the use of a clinical audiometer Interacoustic AD229e and TDH-39 headphones calibrated in accordance with ISO 389-1:1998. The hearing threshold was determined by using the ascending method in accordance with ISO 8253-1:2010. Additionally, based on the pure-tone audiometry, the bilateral hearing threshold was calculated by choosing for each frequency the threshold of the ear that heard better at this particular frequency.

A test-retest analysis of calibration coefficients was conducted, as well as 1-way ANOVA for measurement duration and difficulty. Calibration errors were determined by means of variance estimation. Statistical analyses were performed on the basis of confidence intervals that were estimated in the same way. Estimation of the variance was conducted based on measurement variances and their confidence intervals calculated from the variance and the sample size [16].

## Results

### Test-Retest Analysis

The 25 participants (11 men, 14 women), aged between 22 and 35 years (median 27), who took part in the study completed all the examinations and filled in the questionnaire. All participants were skilled in computer use. On the basis of series 1 and 3, a test-retest analysis was conducted. For each method, a mean difference, standard deviation of the difference and corresponding confidence intervals were calculated (Table 1). Mean values for the dual tone (2 dB), Bekesy (continual), and Bekesy (modulated) methods were significantly different than zero at the significance level of *P*=.05. At *P*=.01, this relation was insignificant for all the methods. The smallest standard deviation was obtained for the Bekesy (modulated) method.

**Table 1:** Mean difference and standard deviation of the difference with corresponding confidence intervals at *P*=.05 of hearing thresholds and calibration coefficients between series 1 and 3 calculated jointly for 8 frequencies (N=25).

Method		Difference (dB), mean (95% CI)	Difference (dB), SD (95% CI)
**Hearing threshold**			
	Right ear	–0.38 (–1.13, 0.38)	5.40 (4.92, 5.99)
	Left ear	0.13 (–0.62, 0.87)	5.34 (4.86, 5.92)
	Both ears	–0.13 (–0.65, 0.40)	5.37 (5.02, 5.77)
	Bilateral	–0.35 (–1.04, 0.34)	4.92 (4.48, 5.46)
**Calibration coefficient**			
	Modulated signal	–0.09 (–0.78, 0.61)	4.97 (4.53, 5.51)
	Dual tone (5 dB)	1.05 (0.00, 2.11)	7.54 (6.87, 8.37)
	Dual tone (2 dB)	1.20 (0.06, 2.35)	8.18 (7.45, 9.07)
	Ascending (5-dB step)	–0.15 (–1.00, 0.70)	6.05 (5.51, 6.71)
	Ascending (2-dB step)	–0.10 (–0.80, 0.60)	5.00 (4.55, 5.54)
	Bekesy (continual)	0.88 (0.19, 1.57)	4.92 (4.48, 5.46)
	Bekesy (modulated)	0.63 (0.09, 1.17)	3.87 (3.52, 4.29)

### Duration of Calibration

**Figure 1:**
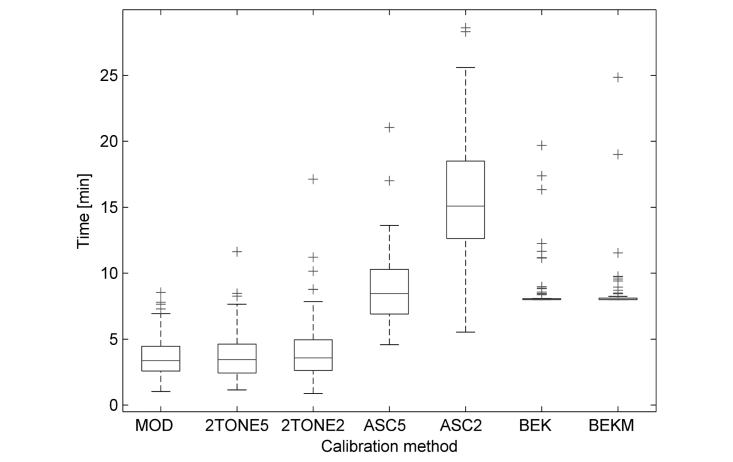
Calibration durations for all 7 calibration methods in series 1-3 (N=25). The horizontal line in each box represents the median, top and bottom box borders represent 75th and 25th percentiles, respectively; crosses represent outliers. MOD: modulated signal; 2TONE5: dual tone (5 dB); 2TONE2: dual tone (2 dB); ASC5: ascending (5-dB step); ACS2: ascending (2-dB step); BEK: Bekesy (continual); BEKM: Bekesy (modulated).

Durations of calibration in relation to the calibration methods are presented in Figure 1. The durations were significantly different (*P*<.001).

The shortest times were obtained for the modulated signal method and both dual tone methods (5 and 2 dB), which consisted in self-adjusting the volume marker. The mean duration of calibration based on the ascending method with the step of 5 dB was comparable to the duration of calibration using both Bekesy methods (continual and modulated). In the Bekesy methods, the outliers are those examinations that were paused momentarily.

### Calibration’s Degree of Difficulty

**Figure 2:**
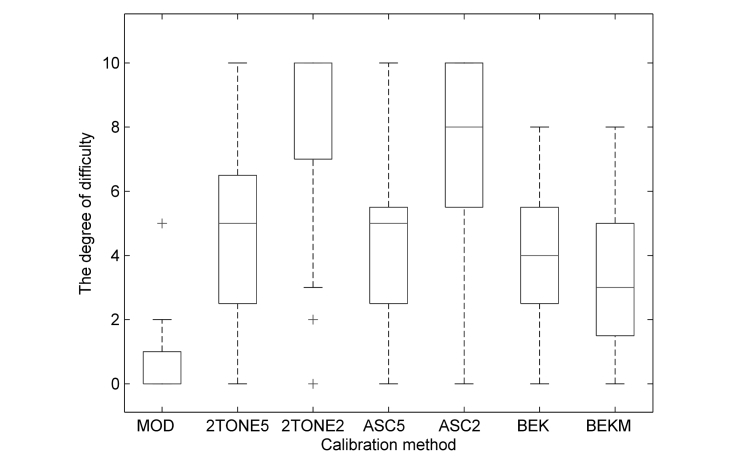
Difficulty ratings of the calibration methods evaluated by 25 participants (0=easiest; 10=hardest). The horizontal line in each box represents the median, top and bottom box borders represent 75th and 25th percentiles, respectively; crosses represent outliers. MOD: modulated signal; 2TONE5: dual tone (5 dB); 2TONE2: dual tone (2 dB); ASC5: ascending (5-dB step); ACS2: ascending (2-dB step); BEK: Bekesy (continual); BEKM: Bekesy (modulated).

The degree of difficulty of the methods were significantly different (*P*<.001). The easiest method of calibration was the modulated signal method consisting in self-adjusting the volume marker in such a way that the presented tone was barely audible. Subsequently, the easiest methods were based on Bekesy’s audiometry (Figure 2).

### Evaluation of the Frequency Response Model

There were 3 methods used to determine the reference sound level. Two were based on the frequency response model of a common sound card and headphones set. Therefore, comparison of methods requires prior evaluation of the model that was conducted using standard deviation of the residual. The mean standard deviation of the residual was calculated on the basis of the differences between the model and the coefficients in series 2 after taking into account the measurement error of coefficients, bilateral hearing threshold of the reference person, and measurement error of this threshold. Measurement error of calibration coefficients and the measurement error of bilateral hearing threshold were calculated from the test-retest differences between series 1 and 3. The standard deviation of the residual estimated in this way describes the difference between the actual coefficients and those calculated for the model fitted on their basis. This standard deviation is independent of the measurement method and the hearing threshold of the reference person. Detailed calculations are presented subsequently. A detailed list of all equations can be found in Multimedia Appendix 2.

Let us assume that *C*_*i*_ is the real value of the calibration coefficient at frequency *f*_*i*_, and *c*_*i*_ denotes its value determined with some error. Moreover, let the model be given as a set of coefficients *M*_*i*_ estimated as follows:

*M*_*i*_=mean(*C*)–FR(*f*_*i*_) (1)

where mean(*C*) is the mean value of coefficients *C*_*i*_ and FR(*f*_*i*_) is the frequency response of the model.

Let’s assume that random variable *X* describes the desired difference between the model *M*_*i*_ and coefficients *C*_*i*_ and random variable *Y* denotes the determination error of the coefficient *c*_*i*_, namely the difference *C*_*i*_–*c*_*i*_. Let’s also define the random variable *Z* as the difference between the model *M*_*i*_ and determined coefficient *c*_*i*_. In this way, we obtain 3 random variables *X*, *Y*, and *Z*, which take on the following values *x*_*i*_, *y*_*i*_, and *z*_*i*_:

*x_i_=M_i_−C_i_* (2)

*y_i_=C_i_−c_i_* (3)

*z_i_=M_i_−c_i_* (4)

It is worth noting that random variables *X* and *Y* are independent. The standard error of the model fitted to the real calibration coefficients does not depend on the determination error of these coefficients. Therefore, bearing in mind that the variance of the sum of 2 independent random variables is the sum of their variances, the desired variance of the random variable *X* is:

variance(*X*)=variance(*Z*)–variance(*Y*) (5)

The mean value of the determined coefficient is close to the mean value of the real coefficient mean(*c*)≈mean(*C*). Then, on the basis of equation 1 we can calculate that *M*_*i*_≈*m*_i_, where *m*_*i*_ is the model estimated on the basis of coefficients *c*_*i*_:

*m*_*i*_=mean(*c*)–FR(*f*_*i*_) (6)

Bearing in mind that *M*_*i*_≈*m*_*i*_, the variance of random variable *Z* may be calculated on the basis of the difference between coefficients *c*_*i*_ and the model *m*_*i*_ estimated on their basis, according to equation 7 (see Multimedia Appendix 2).

Coefficient *c*_*i*_ was determined by subtracting the bilateral hearing threshold from the measured calibration coefficient (equation 8). Therefore, standard deviation of the random variable *Y* expressing the standard error of coefficient *c*_*i*_ depends on the measurement error of calibration coefficient and the measurement error of the bilateral hearing threshold. Both measurement errors were calculated on the basis of the standard deviation of the differences in test-retest examination (equation 9 and Table 1).

*c*=(measured calibration coefficient)–(bilateral hearing threshold) (8)

variance(*Y*) = (calibration method test-retest difference SD)^2^/2+(bilateral threshold test-retest difference SD)^2^/2 (9)

which, on the basis of equations 5, 7, and 9, allows to estimate variance of the random variable *X* determining the model’s error.

**Figure 3:**
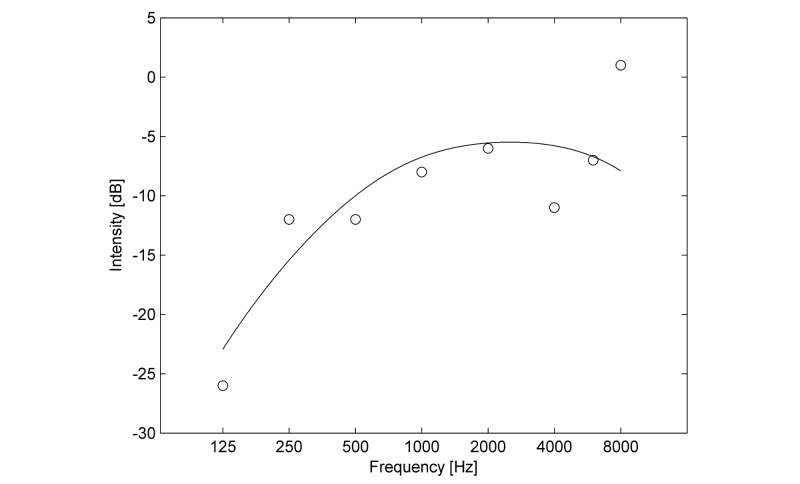
The model of the frequency response fitted to a sample set of calibration coefficients.

Following further calculations, a model based on an A-weight filter was assumed [17] (see equation 10 in Multimedia Appendix 2 and Figure 3).

For each calibration conducted in series 2, variance(*Z*) of the residual of the model was calculated (equation 7), and averaged for every calibration method. Next, for each calibration method, variance(*Y*) was computed on the basis of the standard deviation of the test-retest examination (equation 9 and Table 1). Finally, variance of residual of the model variance(*X*) was estimated independently for each calibration method (equation 5 and Table 2). The mean of standard deviations of residual of the model (model SD 6.57 dB, 95% CI 5.59-7.54) was used for further calculations.

**Table 2:** Standard deviation of residual of the model based on A-weight filter estimated by means of measurements at 8 frequencies carried out by 25 participants.

Calibration method	Model residual (dB), SD
Modulated signal	7.11
Dual tone (5 dB)	6.58
Dual tone (2 dB)	6.36
Ascending (5-dB step)	6.50
Ascending (2-dB step)	7.18
Bekesy (continual)	6.52
Bekesy (modulated)	5.69

### The Reference Sound Level

The error of determining the reference sound level was estimated on the basis of intermediate values: the standard deviation of the bilateral hearing threshold in a population of people with normal hearing, the measurement error of calibration coefficients, and, in the case of methods based on the model, previously calculated error of the model expressed by the standard deviation of the residual. The standard deviation of the bilateral hearing threshold was determined from audiograms after eliminating the assessment error on the basis of the test-retest examination. Measurement error of calibration coefficients was also calculated from a test-retest examination.

The standard deviation of the bilateral hearing threshold measured by the means of pure-tone audiometry is affected by the population variability and measurement error. Knowing, that measurement error is equal to the standard deviation of the bilateral hearing threshold difference in test-retest examination (Table 1) divided by a square root of 2, the standard deviation of the real bilateral hearing threshold can be calculated from equation 11 (Table 3).

(measured bilateral threshold SD)^2^=(real bilateral threshold SD)^2^+(bilateral threshold test-retest difference SD)^2^/2 (11)

**Table 3:** Standard deviation of the bilateral hearing threshold measured in series 1 by 25 participants, estimated measurement error and standard deviation of the real bilateral hearing threshold after eliminating the measurement error with corresponding confidence intervals at *P*=.05.

Frequency		Measured threshold (dB), SD (95% CI)	Measurement error (dB) SE (95% CI)	Real threshold (dB) SD (95% CI)
**Bilateral hearing threshold**				
	At125 Hz	5.58 (4.35, 7.76)	3.48 (3.17,3.86)	4.35 (2.98, 6.73)
	At 250 Hz	5.42 (4.23, 7.53)		4.15 (2.80, 6.47)
	At 500 Hz	4.41 (3.44, 6.13)		2.70 (1.34, 4.76)
	At 1 kHz	3.82 (2.98, 5.31)		1.57 (0.00, 3.65)
	At 2 kHz	4.11 (3.21, 5.72)		2.19 (0.51, 4.22)
	At 4 kHz	5.00 (3.90, 6.96)		3.59 (2.28, 5.79)
	At 6 kHz	6.61 (5.16, 9.20)		5.62 (4.08, 8.35)
	At 8 kHz	7.03 (5.49, 9.78)		6.11 (4.48, 8.98)
	In range 125 Hz-8 kHz	5.36 (4.87, 5.95)		4.07 (3.70, 4.53)
**Mean bilateral hearing threshold**				
	In range 125 Hz-8 kHz	2.62 (2.05, 3.65)	1.23 (1.12,1.37)	2.32 (1.71, 3.38)

The standard deviation of the bilateral hearing threshold difference in test-retest examination was calculated jointly for all frequencies due to lack of significant differences between frequencies in the 1-way ANOVA at the level of statistical significance *P*=.05.

Analogical computation were carried out for the mean value of bilateral hearing threshold, assuming the measurement error divided by a square root of 8 as the mean was for 8 frequencies (Table 3).

The error of the independent coefficients method (determining the reference sound level independently for each frequency on the basis of the calibration coefficient at this frequency) depends on the distribution of the bilateral hearing threshold in the population and the measurement error of the calibration coefficient. Measurement error of the calibration coefficient can be easily calculated from the standard deviation of the difference in the test-retest examination by dividing its value by the square root of 2. Therefore, the mean error of the independent coefficients method across all frequencies may be expressed in the following equation:

(independent coefficients SD)^2^=(real bilateral threshold in the range 125 Hz-8 kHz SD)^2^+(calibration method test-retest difference SD)^2^/2 (12)

where bilateral threshold in the range 125 Hz-8 kHz SD is the standard deviation of the bilateral hearing threshold calculated jointly for all values reduced by the mean at relevant frequencies (Table 3).

The modeled coefficients method consists in estimation of the reference sound level on the basis of the model fitted to the mean value of 8 calibration coefficients determined at various frequencies. Therefore, its error is connected with distribution of the mean bilateral threshold, the error of determining the mean of 8 calibration coefficients, and the standard error of the model. Similarly, as for the independent coefficients method, the error of mean of 8 coefficients can be calculated from the standard deviation of the difference in test-retest examination by dividing its value by the square root of 2, to obtain the error for single coefficient, and by the square root of 8, to obtain the error for the mean. Thus:

(modeled coefficients SD)^2^=(real mean bilateral threshold SD)^2^+(calibration method test-retest difference SD)^2^/16+(model SD)^2^ (13)

Finally, the error of single frequency method consisting in estimating the reference sound level determined on the basis of the model fitted to 1 calibration coefficient at the frequency with the lowest standard deviation will be:

(single frequency SD)^2^=(real bilateral threshold at 1 kHz SD)^2^+(calibration method test-retest difference SD)^2^/2+(model SD)^2^ (14)

The standard errors of each method are presented in Table 4. For practical reasons, the differences in the hearing threshold between measurements on clinical audiometer and biologically calibrated PC were estimated (Table 5). These hearing thresholds were assumed to be obtained by means of ascending methods; therefore, the variances of the calibration methods were increased by the variance of test-retest examination for the ascending method. The variance calculated jointly for both ears was used (Table 1).

**Table 4:** The standard error of biological calibration with corresponding confidence intervals at *P*=.05 estimated on the basis of measurements carried out by 25 participants.

Method	Reference sound level (dB), SE (95% CI)		
	Independent coefficients	Modeled coefficients	Single coefficient
Modulated signal	5.38 (4.89, 5.98)	7.07 (5.95, 8.38)	7.61 (6.35, 9.24)
Dual tone (5 dB)	6.71 (6.10, 7.45)	7.21 (6.09, 8.53)	8.60 (7.32, 10.26)
Dual tone (2 dB)	7.07 (6.43, 7.85)	7.26 (6.13, 8.57)	8.89 (7.60, 10.55)
Ascending (5-dB step)	5.91 (5.37, 6.56)	7.12 (6.00, 8.44)	7.99 (6.73, 9.63)
Ascending (2-dB step)	5.39 (4.90, 5.99)	7.07 (5.95, 8.38)	7.62 (6.36, 9.25)
Bekesy (continual)	5.35 (4.87, 5.95)	7.07 (5.95, 8.38)	7.59 (6.33, 9.23)
Bekesy (modulated)	4.90 (4.46, 5.45)	7.03 (5.91, 8.34)	7.28 (6.02, 8.91)

**Table 5:** The standard deviation of the difference in the hearing threshold determined by means of the ascending method between measurements on clinical audiometer and the biologically calibrated personal computer, together with corresponding confidence intervals at *P*=.05 estimated on the basis of measurements carried out by 25 participants.

Method	Hearing threshold difference (dB), SD (95% CI)		
	Independent coefficients	Modeled coefficients	Single coefficient
Modulated signal	7.60 (7.01, 8.31)	8.88 (7.79, 10.17)	9.31 (8.09, 10.89)
Dual tone (5 dB)	8.59 (7.90, 9.42)	8.99 (7.89, 10.29)	10.14 (8.88, 11.77)
Dual tone (2 dB)	8.88 (8.16, 9.74)	9.03 (7.93, 10.33)	10.38 (9.11, 12.03)
Ascending (5-dB step)	7.98 (7.35, 8.74)	8.92 (7.83, 10.22)	9.63 (8.39, 11.23)
Ascending (2-dB step)	7.61 (7.01, 8.31)	8.88 (7.79, 10.17)	9.32 (8.10, 10.90)
Bekesy (continual)	7.58 (6.99, 8.29)	8.88 (7.78, 10.17)	9.30 (8.08, 10.88)
Bekesy (modulated)	7.27 (6.71, 7.93)	8.84 (7.75, 10.14)	9.05 (7.84, 10.61)

## Discussion

### Principal Findings

This paper presents methods of biological calibration of a PC for hearing examination by determining the reference sound level on the basis of the hearing threshold of the reference person. Seven methods of measuring calibration coefficients and 3 methods of determining reference sound level on the basis of these coefficients were proposed and analyzed. On the basis of 3 series of measurements conducted by 25 participants, the difference between classical pure-tone audiometry and audiometry based on biological calibration was estimated. The smallest standard deviation of the difference was obtained for the Bekesy (modulated) method with the independent coefficients method at the level of 7.27 dB (95% CI 6.71-7.93).

### Comparison of Measurement Methods

The lowest standard deviation in test-retest examination at the level of 3.87 dB (95% CI 3.52-4.29) was obtained using the Bekesy (modulated) method, which entails assessment of the hearing threshold by means of the amplitude-modulated sound according to the rules of Bekesy’s audiometry. This value is in-line with the standard deviation of the test-retest examination of Bekesy’s audiometry [18]. The Bekesy (modulated) method is of moderate duration and is relatively easy to conduct. The modulated signal method, which consists in self-adjusting the volume of the amplitude-modulated sound to the barely audible level, turned out to be the easiest and the quickest method. In the test-retest examination, the standard deviation of this method was 4.97 dB (95% CI 4.53-5.51). The greatest error was found in the dual tone methods consisting in self-adjusting the volume of 2 generated sound signals differing slightly in intensity by a constant value in such a way that only the louder of the 2 sounds was audible.

### Comparison of Sound Reference Level Determination Methods

The estimated error of determining reference sound level turned out to be the lowest for the independent coefficients method and higher for the modeled coefficients method (Table 4). This relation was statistically significant for the modulated signal, ascending (5-dB step), ascending (2-dB step), Bekesy (continual), and Bekesy (modulated) methods (*P*=.05). The highest error occurred for the single frequency method. However, when compared with the modeled coefficients method, statistical significance was achieved only for the dual tone (5 dB) method (*P*=.05).

The standard error of the modeled coefficients method was estimated for the model determined in the frequency range 125 Hz-8 kHz. When the range is limited to 250 Hz-8 kHz, the standard error of the model decreases from 6.57 dB (95% CI 5.59-7.54) to 5.98 dB (95% CI 4.45-7.50). This improves the modeled coefficients method, but the independent coefficients method is still more accurate. However, in this case the relation remained statistically significant only for the Bekesy (modulated) method (*P*=.05).

In the single frequency method, only 1 coefficient is needed to fit the model, which indicates calibration time is 8 times shorter at the cost of higher calibration error.

### Comparison With Previous Work

Some of the presented calibration methods have been used in other studies. In Masalski and Kręcicki [4], calibration was carried out using the dual tone (5 dB) method with single frequency method. The standard deviation of the difference in the hearing threshold between PC-based test and pure-tone audiometry was 10.66 dB, which is in-line with the present study (SD 10.14 dB, 95% CI 8.88-11.77). In Bexelius et al [3], the pure-tone audiometry was compared with the test carried out on a PC calibrated by means of the modulated signal method with independent coefficients method. In all, 89% of the measurements were performed on the same PC and using the same reference person. The standard deviation was obtained at the level of 8.29 dB and 6.9 dB at frequencies 2 kHz and 4 kHz, respectively. These results are also consistent with the present study. The standard deviation for these modulated signal and independent calibration coefficient methods was estimated at the level of 7.60 dB (95% CI 7.01-8.31) (Table 5), whereas if we assume that the reference person is the same by setting the standard deviation of real bilateral threshold in the range 125 Hz-8 kHz to 0 in equation 12, we get 6.42 dB (95% CI 6.13-6.75).

### Other Factors Affecting Accuracy

Calibration error strongly depends on the hearing threshold of the reference person. This applies especially to the independent coefficients and single frequency methods, in which the sound reference level at a single frequency is determined on the basis of a single measurement, contrary to modeled coefficients method, which uses mean hearing threshold. To verify the obtained results, the distribution of the hearing threshold of the participants was compared with literature data (Table 6). The standard deviation of the hearing threshold in this study is significantly smaller (*P*=.01) than the results presented in some studies [19-22], is in-line with one study [23], and is larger than other studies [24-27].

**Table 6:** Summary of standard deviations of the hearing threshold in decibels for participants with normal hearing in the literature.

Study		N	Hearing threshold (dB), SD								Mean SD
			125 Hz	250 Hz	500 Hz	1k Hz	2k Hz	4k Hz	6k Hz	8k Hz	
**Taylor et al, 1967 [24]**											
	18-24 years	46	4.6	4.1	3.9	3.5	3.3	4.1	4.7	4.6	4.1
	25-34 years	33	4.8	4.3	3.9	3.8	4.5	4.3	4.8	5.1	4.4
**Robinson, Sutton, 1979 [19]** ^a^											
	Men	1636	6.6	6.1	5.6	5.6	6.6	7.8	8.9	9.9	7.1
	Women	1578	6.1	5.6	5.6	5.6	6.1	7.2	8.2	9.8	6.8
Arlinger, 1982 [21]		10			7.8	5.2	7.4	6.5	7.4	7.6	7.0
Arlinger, 1991 [25]		30	5.7	5.2	5.0	4.9	4.6	5.1	6.7	6.1	5.4
Lutman, Davis, 1994 [26]		241		4.2	4.4	4.2	4.6	6.9	7.8	7.9	5.7
Han, Poulsen, 1998 [27]		31		4.7	4.4	3.5	4.9	6.6	6.3	7.1	5.4
**Johansson, 2002 [23]** ^b^											
	Men	266	5.7	5.5	5.5	5.4	5.6	6.7	7.0	7.5	6.1
	Women	337	5.5	5.2	4.8	4.5	5.2	7.9	7.4	6.1	5.8
**Engdahl et al, 2005 [22]** ^c^											
	Men	3587		7.6	5.7	5.3	7.0	8.9	9.5	8.0	7.4
	Women	1840		6.3	5.8	5.3	5.7	7.0	9.1	7.6	6.7
Current study		25	5.9	6.1	5.0	4.2	4.9	5.6	8.0	9.1	6.1

^a^Estimated on the basis of the model for the age of 25 years, consistent with ISO 7029, 2000 [20].

^b^Estimated on the basis of centiles calculated from the model for the age of 25 years.

^c^Estimated on the basis of centiles in the age range 20-29 years.

The examinations in this study were conducted on young employees and interns of the Otolaryngology Clinic (ie, persons familiar with the subject of hearing examinations). This may have led to better calibration results and shorter duration of the examination than in a population of young people with good hearing without experience with hearing examinations.

In the calculations, it was assumed that the examinations conducted on home computers are not burdened with an error resulting from the presence of background noises other than the fan noise. This assumption was made because during home examinations and in those conducted in the sound booth the fan noise was the loudest and the most disturbing sound. Thus, the estimated calibration error takes into account the fan noise. However, in the case of other background noises, the error may turn out to be bigger.

Calibration methods presented in the paper were implemented as Java applets embedded in browsers. However, their application is not limited only to Web-based tests, but may also be used for offline determination of the reference sound level or on mobile devices. Moreover, in the case of tablets or smartphones, calibration error may turn out to be smaller because of the lack of fan noises.

When conducting examinations on a PC with the use of headphones with very high sensitivity instead of regular ones, interferences of the sound card or other electronic systems may affect the stimulus. During examination at home, such incidents occurred in 2 of 25 cases. As a result, it was impossible to perform the examination. After changing headphones from professional to regular ones, the examination was completed without any problems.

Calibration accuracy may be improved if it is conducted by 2 or more reference persons [3]. The greatest improvement may be expected in the case of the independent coefficients method, whose standard deviation should reduce proportionally to the square root of the number of persons conducting calibration. In the case of the modeled coefficients and single frequency methods, the improvement will be less visible because increasing the number of reference persons does not affect the model’s error.

Another method of improving the accuracy is to introduce additional conditions to reject inaccurate calibrations. For example, calibration using the independent coefficients method may be rejected as the difference between coefficients exceeds the predetermined threshold [3]. In the case of the modeled coefficients method, the condition may be imposed on the difference between the value of the calibration coefficient and the model. In the single frequency method, it is possible to do an additional measurement and verify its value with the model. Moreover, for all methods based on Bekesy’s audiometry, verification can be based on the difference between the intensities at which the sound starts to be audible and the intensities at which the sound ceases to be audible.

### Recommendations

The final choice of the calibration method will depend on the desired accuracy of calibration and the time for its performance. If considerable accuracy is required, it is advisable to use the independent coefficients method, whereas when quick calibration is the priority, the single frequency method is preferable. The application of the modeled coefficients method is not justified because of higher calibration error than is in the independent coefficients method at the same duration.

Two of the 7 methods of measuring calibration coefficients seem worth noting: the modulated signal and Bekesy (modulated) methods. The choice of the better of the 2 is not obvious. The Bekesy (modulated) method is the most accurate at moderate duration, whereas the modulated signal method is the fastest at moderate accuracy. Additionally, the modulated signal method is the easiest, and the Bekesy (modulated) method is the second easiest. However, the methods differ significantly in the complexity of implementation with the Bekesy (modulated) method being more complex. On the other hand, in the case of Bekesy (modulated) method, the measurement can be easily verified on the basis of the differences between the intensities at which the stimulus starts or stops being audible.

Therefore, if there are no substantial time limitations, it is advisable to use Bekesy (modulated) method with independent coefficients method, which have the lowest error. When a simple and quick calibration is required, modulated signal method with single frequency method should be chosen.

## Multimedia Appendix 1

Translation of the questionnaire on difficulty of the calibration.

## Multimedia Appendix 2

All equations used for this paper: (1) the model, (2) the difference between the model and the real calibration coefficient, (3) the determination error of the calibration coefficient, (4) the difference between the model and the determined coefficient, (5) the variance of random variable X, (6) the model estimated on the basis of determined coefficients, (7) the variance of random variable Z, (8) the value of determined coefficient, (9) the variance of random variable Y, (10) the frequency response of the model, (11) the standard deviation of the participants' bilateral hearing threshold measured with ascending method, (12) the standard error of the independent coefficients method, (13) the standard error of the modeled coefficients method, (14) the standard error of the single frequency method.

## Multimedia Appendix 3

CONSORT-EHEALTH checklist V1.6.2 [28].

## Abbreviations

dB: decibel
dB HL: decibel hearing level
PC: personal computer
