# Supplementary material for: Biological Calibration for Web-Based Hearing Tests: Evaluation of the Methods
Source: J Med Internet Res. 2014 Jan 15;16(1):e11. doi: 10.2196/jmir.2798 (PMC3906690; doi:10.2196/jmir.2798)
Supplement: Supplementary file 1 [file jmir_v16i1e11_app1.pdf]

The Jan Mikulicz-Radecki University Research and Teaching Hospital in Wrocław
Department and Clinic of Otolaryngology, Head and Neck Surgery, Wroclaw Medical University
Hospital Director: prof. dr hab. Tomasz Kręcicki
50-556 Wrocław, ul Borowska 213

Questionnaire for participants
BIOLOGICAL CALIBRATION FOR WEB-BASED HEARING TESTS
EVALUATION OF THE METHODS

Name: ………………………………………………

Evaluate the calibration methods in terms of their difficulty (0 – the easiest method, 10 – the most difficult method):

_____ Calibration with a sinusoidal signal. During the calibration the slider hast to be moved in such a way that the signal is on the verge of audibility.
_____ Calibration with two signals differing by 5dB. During the calibration the slider has to be adjusted so that only the louder sound is audible.
_____ Calibration with two signals differing by 2 dB. During the calibration the slider has to be adjusted so that only the louder sound was audible.
_____ Calibration with the ascending method with the step of 5 dB. When the signal is audible the button has to be held for as long as it is audible.
_____ Calibration with the ascending method with the step of 2 dB. When the signal is audible the button has to be held for as long as it is audible.
_____ Calibration with frequency changing signal. When the signal is audible the button has to be held for as long as it is audible.
_____ Calibration with a modulated frequency changing signal. When the signal is audible the button has to be held for as long as it is audible.
