# Supplementary figures and images for: Biological Calibration for Web-Based Hearing Tests: Evaluation of the Methods
Source: J Med Internet Res. 2014 Jan 15;16(1):e11. doi: 10.2196/jmir.2798 (PMC3906690; doi:10.2196/jmir.2798)

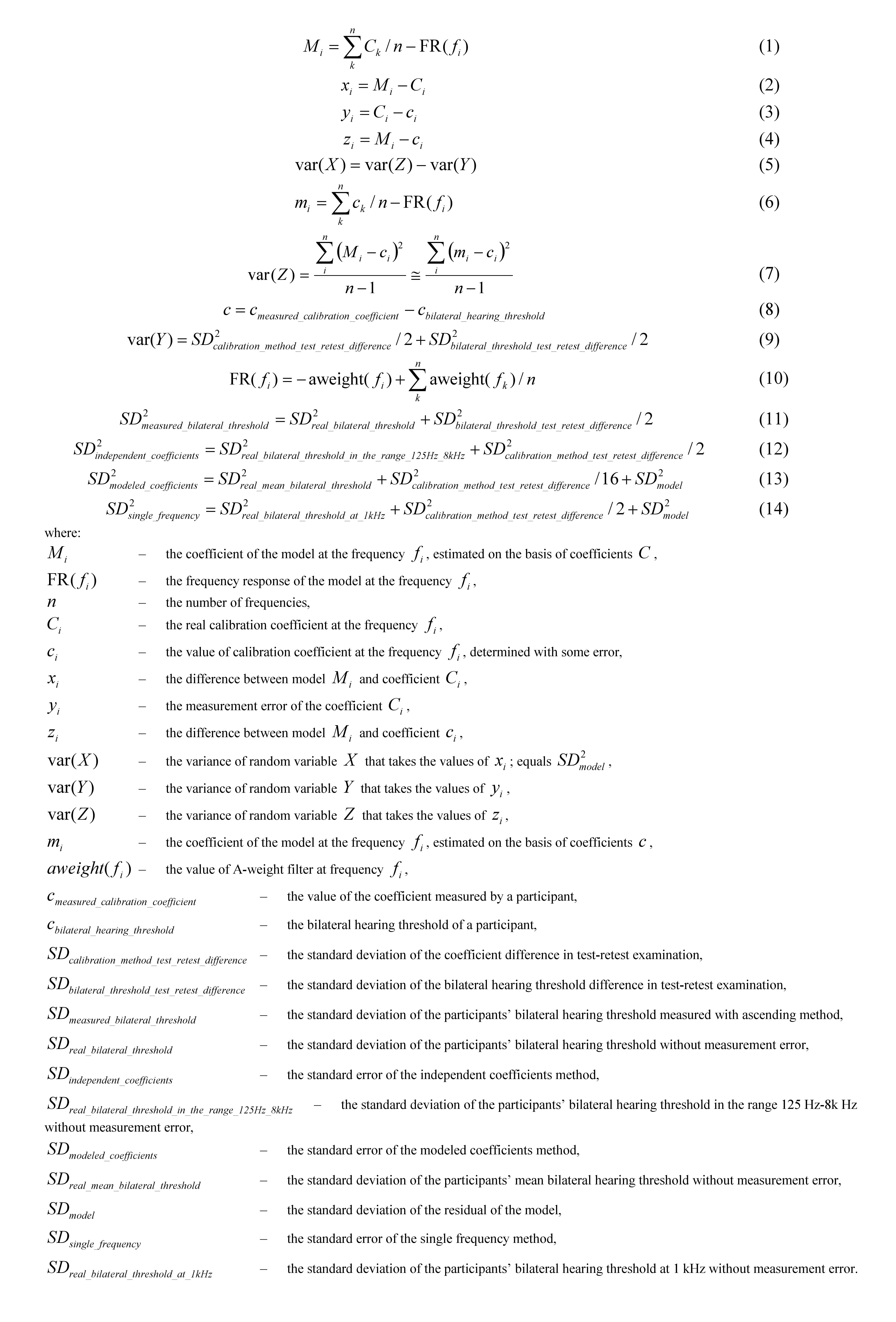

Supplement: Supplementary file 2 [file jmir_v16i1e11_app2.png]
