# Supplementary material for: Biological Calibration for Web-Based Hearing Tests: Evaluation of the Methods
Source: J Med Internet Res. 2014 Jan 15;16(1):e11. doi: 10.2196/jmir.2798 (PMC3906690; doi:10.2196/jmir.2798)
Supplement: Supplementary file 3 [file jmir_v16i1e11_app3.pdf]

CONSORT-EHEALTH Checklist V1.6.2 Report

(based on CONSORT-EHEALTH V1.6), available at [http://tinyurl.com/consort-ehealth-v1-6].

2798

Date completed

10/9/2013 7:32:30

by

Marcin Masalski

Biological Calibration for Web-Based Hearing Tests: Evaluation of the Methods

TITLE

1a-i) Identify the mode of delivery in the title

Methods presented in the article were assessed on the basis of applets embedded in a search engine. Thus, the expression “Web-based” in the title of the paper. In "Discussion" it was stressed that they may also be used in offline and mobile tests.

"Calibration methods presented in the paper were implemented as Java applets embedded in browsers. However, their application is not limited only to web-based tests, but may also be used for offline determination of the reference sound level or on mobile devices. Moreover, in the case of tablets or smartphones, calibration error may turn out to be smaller due to the lack of fan noises."

1a-ii) Non-web-based components or important co-interventions in title

The difficulty of calibration methods was assessed in an offline questionnaire. The significance of the questionnaire was secondary and therefore it was not mentioned in the title. In paragraph 6 of "Methods" the word "offline" was added next to the word "questionnaire".

1a-iii) Primary condition or target group in the title

Studies of calibration were conducted on a group of people with normal hearing. This results directly from the idea of biological calibration and thus mention of this group in the title seems excessive.

ABSTRACT

1b-i) Key features/functionalities/components of the intervention and comparator in the METHODS section of the ABSTRACT

Key features: "Seven methods have been proposed for measuring the calibration coefficients. All measurements were performed in reference to the hearing threshold of the normal hearing person. Then, three methods were proposed for determining the reference sound level on the basis of these calibration coefficients."

Comparator: "Methods were compared in terms of the estimated error, duration and difficulty of the calibration."

1b-ii) Level of human involvement in the METHODS section of the ABSTRACT

"Web-based self-assessed measurements of the calibration coefficients were carried out" as well as "pure-tone audiometry" and "offline questionnaire concerning the difficulty of the calibration".

1b-iii) Open vs. closed, web-based (self-assessment) vs. face-to-face assessments in the METHODS section of the ABSTRACT

The trial was closed ("Participants were recruited offline from co-workers of the Department and Clinic of Otolaryngology."), self-assessed ("Web-based self-assessed measurement of the calibration coefficients…') with face-to-face ("clinical pure-tone air-conducted audiometry") and offline ("offline questionnaire") components.

1b-iv) RESULTS section in abstract must contain use data

"All of the 25 participants, aged 22-35, median 27 completed the all the tests and filled in the questionnaire."

The estimated error of calibration coefficients for the most accurate method was presented ("The smallest standard deviation of the calibration coefficient in the test-retest measurement was obtained at the level of 3.87dB (95% CI 3.52dB-4.29dB)...") as well as for the easiest and the quickest ("The simplest and shortest turned out to be the method of self-adjustment of the sound volume to the barely audible level (MOD). In the test-retest measurement the deviation of this method equaled 4.97dB (95% CI 4.53dB-5.51dB). ").

The most accurate method of determining the reference sound level was indicated ("Among methods determining the reference sound level, the levels determined independently for each frequency were burdened with the smallest error ...") and the estimated range of errors between the examination based on biological calibration and pure-tone audiometry were provided ("from 7.27dB (95% CI 6.71dB-7.93dB) to 10.38dB (95% CI 9.11dB-12.03dB), depending on the calibration method.").

1b-v) CONCLUSIONS/DISCUSSION in abstract for negative trials

The item is not relevant for this study. ("The objective of the study was to identify the error of the proposed methods of biological calibration, the assessment of their duration and the subjective difficulty in its conducting.")

INTRODUCTION

2a-i) Problem and the type of system/solution

Problem: "... most hearing tests (ed. conducted remotely in home settings on PCs), including the basic examination in the form of pure-tone audiometry, require prior calibration of the system..."

Solution and goals of the intervention: "... Both the above-mentioned solutions limit accessibility of the hearing test as they require efforts which are unjustified in the case of a single hearing test. In the light of the above, biological calibration seems a sensible solution..."

Patient population: "... it may be applied as a screening tests as well as in other situations suggested in papers [3,4], i.e. self-monitoring of the hearing in case of disorders such as fluctuating hearing loss, tinnitus, sudden deafness, otosclerosis, Ménière's disease, during treatment with ototoxic drugs, in large-scale epidemiological studies, in the case of limited access to specialist equipment e.g. at the GP or in countries with low economic status, and also as a telemedical examination combined with a questionnaire whose aim is to determine the direction of further treatment."

2a-ii) Scientific background, rationale: What is known about the (type of) system

What is known about the (type of) system: "Honeth et al [3] used biological calibration... The test results were compared with pure-tone audiometry and exhibited... Masalski and Kręcicki [4] also used biological calibration... Self-examinations... showed a mean error of the hearing threshold compared to pure-tone audiometry at the level of -1.35±10.66dB... The error analysis of the pure-tone audiometry conducted on a PC calibrated by means of the biological method showed significant influence of the calibration error [4]."

Motivation for the study: "The application of pure-tone audiometry based on biological calibration depend significantly on the measurement error... prior to verification of these applications it is advisable to optimize biological calibration [4]."

Choice of comparator: The measurement error, the measurement time and the difficulty are common and important features of the calibration method.

METHODS

3a) CONSORT: Description of trial design (such as parallel, factorial) including allocation ratio

Specific objectives are explicitly stated in the abstract: "The objective of the study was to identify the error of the proposed methods of biological calibration, the assessment of their duration and the subjective difficulty in its conducting."
In "Introduction" they were described in more detail: "This paper presents seven methods of measuring the calibration coefficients. All measurements were performed in reference to the hearing threshold of the normal hearing person. For each method the measurement error was determined, as well as timeframe for its calibration and the difficulty level. Next, three methods were proposed for determining the reference sound level on the basis of these calibration coefficients and for each of them an error analysis was conducted."

3b) CONSORT: Important changes to methods after trial commencement (such as eligibility criteria), with reasons

Once the examinations were started no important changes were introduced.

3b-i) Bug fixes, Downtimes, Content Changes

Due to lack of connection calibration coefficients for BEK method were not recorded. This was a single incident. The examination was repeated.

4a) CONSORT: Eligibility criteria for participants

"The eligibility criteria were age under 35, lack of previous hearing problems, owning headphones and a PC at home, basic skills to operate it, and the willingness to participate in the research."

4a-i) Computer / Internet literacy

"... basic skills to operate it..."
Moreover, in "Results" section: "All participants were skilled in computer use".

4a-ii) Open vs. closed, web-based vs. face-to-face assessments:

"Participants were recruited offline from co-workers of Otolaryngology Clinic using face-to-face prompting."

4a-iii) Information giving during recruitment

During an interview co-workers were asked to take part in the study. They were informed about the study objectives, the conditions of its conducting and its duration (see Multimedia Appendix Information for the research participants).

4b) CONSORT: Settings and locations where the data were collected

"Each participant performed calibration using all seven methods three times: in series (i) the study was carried out in the soundbooth with the use of notebook Dell Vostro 1310 with Microsoft Windows 7 operational system and Technics RP-F290 headphones, in series (ii) each person was asked to perform calibration on their own home computer using their own headphones in possibly quiet conditions, preferably late in the evening or at night to minimize background noise level and to create conditions close to those prevailing in soundbooth, and series (iii) was the repetition of examinations from series (i). "
"Moreover, in series (i) and (iii) a pure-tone audiometry was performed with the use of clinical audiometer Interacoustic AD229e and TDH-39 headphones calibrated in accordance with ISO 389-1:1998. The hearing threshold was determined using the ascending method, in accordance with ISO 8253-1:2010. "

4b-i) Report if outcomes were (self-)assessed through online questionnaires

Online questionnaire was not used.
"On completion of all the tests the subject filled in a offline questionnaire on the difficulty of the tests by assigning each method values from 0 (the easiest method) to 10 (the hardest one)."

4b-ii) Report how institutional affiliations are displayed

The affiliations were not displayed. However, the participants were recruited from among Clinic workers and the affiliation was known to them.

5) CONSORT: Describe the interventions for each group with sufficient details to allow replication, including how and when they were actually administered

5-i) Mention names, credential, affiliations of the developers, sponsors, and owners

"The first author of this article is the owner of the Internet portal e-audiologia.pl that offers online hearing tests."
"The research described in this article had been carried out as part of a project Kluczowy Stażysta no KSW/13/I/2011 co-financed from the European Social Fund."

5-ii) Describe the history/development process

"All the seven methods were implemented in Java technology in the form of applets embedded in a web browser".
Fragments of the source code created for the purposes of e-audiologia.pl website offering online hearing tests were used. Applets were prepared and tested by the authors of the paper.

5-iii) Revisions and updating

No changes were made during examinations.

5-iv) Quality assurance methods

Software testing before trial started.

5-v) Ensure replicability by publishing the source code, and/or providing screenshots/screen-capture video, and/or providing flowcharts of the algorithms used

Screenshots of the website on the bases of which the trial was conducted were added in Multimedia Appendices.

5-vi) Digital preservation

Website used in the research was archived in a context "JMIRms2798":
http://www.e-audiologia.pl/JMIRms2798/test324567875/testy.jsp?id=2798&dla=JMIRms2798
and in webcitation.org:
http://www.webcitation.org/6KE0Vb9fZ

5-vii) Access

Each participant received three links prepared especially for them, one for each examination series. The link led to the list of examinations and from there directly to the examination, i.e. a website with a Java applet (compare 5-vi and 5-v).

5-viii) Mode of delivery, features/functionalities/components of the intervention and comparator, and the theoretical framework

Mode of delivery: series (i) and (iii): direct, series (ii): email with link to webpage.
Instructional strategy: face-to-face interview and online instructions.During recruitment, participants who are employees of the Otolaryngology Clinic were informed about examination objectives, conditions of its conducting and its duration (see Multimedia Appendix "A leaflet for participants"). Detailed information concerning each method were presented on the website directly under each measurement (see 5-v and 5-vi). Additionally "each person was asked to perform calibration on their own home computer using their own headphones in possibly quiet conditions, preferably late in the evening or at night to minimize background noise level and to create conditions close to those prevailing in sound booth".
Delivery platform: series (i) and (iii): "notebook Dell Vostro 1310 with Microsoft Windows 7 operational system and Technics RP-F290 headphones", series (ii): "…each person performed calibration on their own home computer using their own headphones…"
Description of the content:
- page design principles, average amount of text on pages, presence of hyperlinks to other resources: see Multimedia Appendix "Screenshots of a website",
- the whole application was developed by the authors,
- the application was not intended to allows users to track results of previous examinations.
Description of communication delivery channels: computer-mediated communication is not a component.

5-ix) Describe use parameters

"Each participant performed calibration using all seven methods three times: in series (i) the study was carried out in the soundbooth with the use of notebook Dell Vostro 1310 with Microsoft Windows 7 operational system and Technics RP-F290 headphones, in series (ii) each person was asked to perform calibration on their own home computer using their own headphones in possibly quiet conditions, preferably late in the evening or at night to minimize background noise level and to create conditions close to those prevailing in soundbooth, and series (iii) was the repetition of examinations from series (i)."

5-x) Clarify the level of human involvement

The instruction for conducting measurements were clear to the participants. They were, however, persons familiar with hearing tests. While measurements were conducted no assistance was needed.

5-xi) Report any prompts/reminders used

No evidence of the number of reminders about the examination was recorded, as the significance of such evidence was secondary. Most participants performed examination after 2-3 reminders during a conversation. Reminders about examinations in series (ii) were sent by email.

5-xii) Describe any co-interventions (incl. training/support)

No co-interventions were provided.

6a) CONSORT: Completely defined pre-specified primary and secondary outcome measures, including how and when they were assessed

Despite the fact that the research is not Randomized Controlled Trial, primary outcome measures can be assumed to be the error of the calibration method, whereas secondary – its duration and difficulty.

6a-i) Online questionnaires: describe if they were validated for online use and apply CHERRIES items to describe how the questionnaires were designed/deployed

No online questionnaires were used.
The questionnaire concerning the difficulty of calibration methods was translated and is available in Multimedia Appendix (see Multimedia Appendix "Translation of the questionnaire on difficulty of the calibration").

6a-ii) Describe whether and how "use" (including intensity of use/dosage) was defined/measured/monitored

"The calibration coefficients expressing the sound intensity in decibels, together with the duration of examinations were recorded in the database."

6a-iii) Describe whether, how, and when qualitative feedback from participants was obtained

Because research participants are the authors' co-workers the feedback concerning problems encountered while conducting the examination was provided in the oral form. The participants reported concerning the ventilation noise and twice problems connected with headphones. These problems were discussed in "Discussion" (see point 19) item (1) and (2).

6b) CONSORT: Any changes to trial outcomes after the trial commenced, with reasons

No changes to outcomes were introduced during the trial.

7a) CONSORT: How sample size was determined

7a-i) Describe whether and how expected attrition was taken into account when calculating the sample size

If we assume the value of measurement error in pure-tone audiometry conducted on the basis of biological calibration obtained in the previous works at the level of 9.78dB [4] and the expected confidence interval at the level of +-1dB at statistical significance P=.05, we get the number of participants of 25 for measurements at 8 frequencies (number of measurements N=25*8, the degrees of freedom N-8, for the assumed error of 9.78dB, 95% CI is between 8.89 and 10.87, which results in the interval width of 1.98dB)

7b) CONSORT: When applicable, explanation of any interim analyses and stopping guidelines

Indirect analyses were conducted in order to confirm that the sample size is appropriate.

8a) CONSORT: Method used to generate the random allocation sequence

Not applicable – no randomization.

8b) CONSORT: Type of randomisation; details of any restriction (such as blocking and block size)

Not applicable – no randomization.

9) CONSORT: Mechanism used to implement the random allocation sequence (such as sequentially numbered containers), describing any steps taken to conceal the sequence until interventions were assigned

Not applicable – no randomization.

10) CONSORT: Who generated the random allocation sequence, who enrolled participants, and who assigned participants to interventions

Not applicable – no randomization.

11a) CONSORT: Blinding - If done, who was blinded after assignment to interventions (for example, participants, care providers, those assessing outcomes) and how

11a-i) Specify who was blinded, and who wasn't

The conducted study was not RCT. However:
The participants were informed about the measurement method. Statistical analysis was conducted by one person (the first author of this paper), who was also aware of the measurement method.

11a-ii) Discuss e.g., whether participants knew which intervention was the "intervention of interest" and which one was the "comparator"

As above, the participants were aware of the measurement method and the objective of the conducted examination.

11b) CONSORT: If relevant, description of the similarity of interventions

The conducted study was not RCT. However:
All measurement methods were near threshold measurements, and within one series they were conducted in the same conditions and using the same equipment.

12a) CONSORT: Statistical methods used to compare groups for primary and secondary outcomes

"A test-retest analysis of calibration coefficients was conducted, as well as one-way ANOVA for measurement duration and its difficulty. Calibration errors were determined by means of variance estimation. Statistical analysis was performed on the basis of confidence intervals that were estimated in the same way. Estimation of the variance was conducted based on measurement variances and their confidence intervals calculated from the variance and the sample size [16]."

12a-i) Imputation techniques to deal with attrition / missing values

"All 25 participants (11 men, 14 women), aged 22-35, median 27, who took part in the study completed all the examinations and filled in the questionnaire."

12b) CONSORT: Methods for additional analyses, such as subgroup analyses and adjusted analyses

No subgroup or adjusted analyses were performed.

RESULTS

13a) CONSORT: For each group, the numbers of participants who were randomly assigned, received intended treatment, and were analysed for the primary outcome

The conducted study was not RCT. However:
“All of the 25 participants, aged between 22-35, median 27 who took part in the study completed all examinations and filled out the questionnaire.” All results were analysed.

13b) CONSORT: For each group, losses and exclusions after randomisation, together with reasons

The conducted study was not RCT. However:
“All of the 25 participants … completed all examinations and filled out the questionnaire.” See also "The trial design and the realization" diagram in Multimedia Appendices.

13b-i) Attrition diagram

After completing the study the measurements are no longer conducted.

14a) CONSORT: Dates defining the periods of recruitment and follow-up

The measurements were conducted from 01.09.2012 to 31.03.2013

14a-i) Indicate if critical “secular events” fell into the study period

No "secular events" occurred.

14b) CONSORT: Why the trial ended or was stopped (early)

The experiment was finished after the intended number of participant had been examined.

15) CONSORT: A table showing baseline demographic and clinical characteristics for each group

The description was included in the text of the paper, not in the table. Compare 15-i.

15-i) Report demographics associated with digital divide issues

"All 25 participants (11 men, 14 women), aged 22-35, median 27... All participants were skilled in computer use". 16 participants had higher education, 9 secondary education.

16a) CONSORT: For each group, number of participants (denominator) included in each analysis and whether the analysis was by original assigned groups

16-i) Report multiple “denominators” and provide definitions

Sample size was given in tables and figures:
Table 1: "...calculated on the basis of measurements conducted by 25 subjects at 8 frequencies."
Figure 1: "...carried out by 25 subjects in series (i-iii)"
Figure 2: "...evaluated by 25 subjects"
Table 2:”... by means of 8 measurement methods carried out by 25 subjects"
Table 3: "... by 25 subjects, ... calculated by means of measurements at 8 frequencies carried out by 25 subjects"
Table 4: "... by 25 subjects"
Table 5: „…estimated on the basis of measurements carried out by 25 subjects.”
Table 6: “…estimated on the basis of measurement carried out by 25 subjects.”

16-ii) Primary analysis should be intent-to-treat

The conducted study was not RCT. Compare 16-i.

17a) CONSORT: For each primary and secondary outcome, results for each group, and the estimated effect size and its precision (such as 95% confidence interval)

Statistical description of the duration and the degree of difficulty was presented on the box plots. Calibration error and indirect values needed for its calculation were given together with 95% confidence interval.

17a-i) Presentation of process outcomes such as metrics of use and intensity of use

(1) Calibration duration was measured from the start to the finish of the Java applet.
(2) "… the subject filled in a offline questionnaire on the difficulty of the tests by assigning each method values from 0 (the easiest method) to 10 (the hardest one)." See Multimedia Appendix "Translation of the questionnaire on difficulty of the calibration".
(3) The method of estimation of calibration error consists of several steps and was presented in "Results".

17b) CONSORT: For binary outcomes, presentation of both absolute and relative effect sizes is recommended

There are no binary outcomes in the paper.

18) CONSORT: Results of any other analyses performed, including subgroup analyses and adjusted analyses, distinguishing pre-specified from exploratory

No subgroup analyses nor adjusted analyses were performed.

18-i) Subgroup analysis of comparing only users

No subgroup analyses nor adjusted analyses were performed.

19) CONSORT: All important harms or unintended effects in each group

(1) Research participants drew attention to the fact that both in the soundbooth, as well as at home the computer fan, working at certain intervals, casued significant disturbances during measurements. The error connected with the noise of computer fan is a part of the specificity of the computer-based measurement and was included in the estimated calibration error.
(2) When conducting examinations on a PC with the use of headphones with very high sensitivity instead of regular ones, interferences of the sound card or other electronic systems may affect the stimulus. During examination at home such incidents occurred in 2 out of 25 cases. As a result, it was impossible to perform the examination. After changing headphones from professional to regular ones the examination was completed without any problems.
(3) During measurement using BEK method at audiology outpatient clinic in the case of one of 25 persons human error occurred. The difference between calibration coefficients at two different frequencies was about 50dB. The examination was repeated.
(4) Due to lack of Internet connection the calibration coefficients of BEK method were not recorded. This was a single incident. The examination was repeated (see 3b-i).

19-i) Include privacy breaches, technical problems
No privacy breaches occured. Technical problems: fan noise and disturbances in headphones (see 19) and lack of Internet connection (see 3b-i).
19-ii) Include qualitative feedback from participants or observations from staff/researchers
Qualitative feedback: fan noise and disturbances in headphones (see 19) and lack of Internet connection (see 3b-i).

DISCUSSION

20) CONSORT: Trial limitations, addressing sources of potential bias, imprecision, multiplicity of analyses
20-i) Typical limitations in ehealth trials
(1) "The examinations were conducted on young employees and interns of the Otolaryngology Clinic, that is persons who are familiar with the subject of hearing examinations. It may lead to better calibration results, and above all, shorter duration of the examination than in the population of young people with good hearing who had not had anything to do with hearing examinations."
(2) "Calibration error strongly depends on the hearing threshold of the reference person. This applies especially to COEFF and MODEL_1 methods, in which the sound reference level at single frequency is determined on the basis of a single measurement, contrary to MODEL_8, which uses mean hearing threshold. In order to verify the obtained results, the distribution of the hearing threshold of the subjects participating in the study was compared with literature data (Table 7)."
(3) "In the calculations it was assumed that the examinations conducted on home computers are not burdened with an error resulting from the presence of background noises other than the fan noise. This assumption was made because during home examinations, as well as those conducted in the soundbooth the fan noise was the loudest and the most disturbing sound. Thus, the estimated calibration error takes into account the fan noise. However, in the case of other background noises the error may turn out to be bigger."
(4) "When conducting examinations on a PC with the use of headphones with very high sensitivity instead of regular ones, interferences of the sound card or other electronic systems may affect the stimulus. During examination at home such incidents occurred in 2 out of 25 cases. As a result, it was impossible to perform the examination. After changing headphones from professional to regular ones the examination was completed without any problems."
21) CONSORT: Generalisability (external validity, applicability) of the trial findings
21-i) Generalizability to other populations
"The examinations were conducted on young employees and interns of the Otolaryngology Clinic, that is persons who are familiar with the subject of hearing examinations. It may lead to better calibration results, and above all, shorter duration of the examination than in the population of young people with good hearing who had not had anything to do with hearing examinations."
21-ii) Discuss if there were elements in the RCT that would be different in a routine application setting
(1) "Due to relatively long duration of the series the subjects were informed about the possibility of taking a break when they felt tired, and the majority of the subjects took advantage of this possibility." However, numerous assessment of near threshold sounds may be boring and lead to worse results.
(2) "..., in the case of tablets or smartphones, calibration error may turn out to be smaller due to the lack of fan noises."
22) CONSORT: Interpretation consistent with results, balancing benefits and harms, and considering other relevant evidence
22-i) Restate study questions and summarize the answers suggested by the data, starting with primary outcomes and process outcomes (use)
"This paper presents methods of biological calibration of a PC for hearing examination which consists in determining the reference sound level on the basis of the hearing threshold of the reference person. Seven methods of measuring calibration coefficients and three methods of determining reference sound level on the basis of these coefficients were proposed and analysed. On the basis of three measurement series conducted by 25 participants the difference between classical pure-tone audiometry and audiometry based on biological calibration was estimated. The smallest standard deviation of the difference was obtained for BEKM+COEFF methods at the level of 7.27dB (95% CI 6.71dB-7.93dB)."
22-ii) Highlight unanswered new questions, suggest future research
(1) Calibration error on electronic equipment which do not have a fan (smartphones, tablets) may be smaller. "Calibration methods presented in the paper were implemented as Java applets embedded in browsers. However, their application is not limited only to web-based tests, but may also be used for offline determination of the reference sound level or on mobile devices. Moreover, in the case of tablets or smartphones, calibration error may turn out to be smaller due to the lack of fan noises."
(2) Calibration error will be smaller if calibration is conducted by more than one person." Calibration accuracy may be improved if it is conducted by two or more reference persons [3]. The greatest improvement may be expected in the case of the COEFF method, whose standard deviation should reduce sqrt(N) times, where N is the number of persons conducting calibration. In the case of the MODEL_8 and MODEL_1 methods the improvement will be less visible as increase in the number of reference persons does not affect the model's error."

Other information

23) CONSORT: Registration number and name of trial registry
The examination was registered with Department of Science of Wroclaw Medical University under the number: BW42.
24) CONSORT: Where the full trial protocol can be accessed, if available
Full trial protocol is not available.
25) CONSORT: Sources of funding and other support (such as supply of drugs), role of funders
"The research described in this article had been carried out as part of a project Kluczowy Stażysta no KSW/13/I/2011 co-financed from the European Social Fund."
X26-i) Comment on ethics committee approval
Consent of Bioethics Committee to conduct the trial has been obtained.
x26-ii) Outline informed consent procedures
"Participants were recruited offline from co-workers of Otolaryngology Clinic using face-to-face prompting."
The translation of the consent form is available in the Multimedia Appendices ("Translation of a leaflet for participants").
X26-iii) Safety and security procedures
See the translation of the consent form in the Multimedia Appendices ("Translation of a leaflet for participants"):
"The tests are painless, non-invasive, and do not pose a threat to a person's hearing as the generated sounds are very low (near hearing threshold)."
X27-i) State the relation of the study team towards the system being evaluated
"The first author of this article is the owner of the Internet portal e-audiologia.pl that offers online hearing tests."
